# A case of simultaneous pancreatoduodenectomy and living donor liver transplantation for biliary cancer complicated with congenital biliary dilatation

**DOI:** 10.1186/s40792-024-02068-5

**Published:** 2024-12-04

**Authors:** Tsuyoshi Shimamura, Masaaki Watanabe, Yasuyuki Koshizuka, Ryoichi Goto, Norio Kawamura, Tatsuya Orimo, Hirofumi Kamachi, Toshiya Kamiyama, Tomoko Mitsuhashi, Taizo Hibi, Akinobu Taketomi

**Affiliations:** 1 Division of Organ Transplantation, Hokkaido University Hospital, Sapporo, Japan; 2 Department of Transplant Surgery, Faculty of Medicine and Graduate School of Medicine, Hokkaido University, Sapporo, Japan; 3 Department of Gastroenterological Surgery I, Graduate School of Medicine, Hokkaido University, Sapporo, Japan; 4 Department of Surgical Pathology, Hokkaido University Hospital, Sapporo, Japan; 5 Department of Pediatric Surgery and Transplantation, Graduate School of Medical Sciences, Kumamoto University, Kumamoto, Japan

**Keywords:** Biliary cancer, Congenital biliary dilatation, Living donor liver transplantation

## Abstract

**Background:**

In patients with pancreaticobiliary maljunction complicated by congenital biliary dilatation, the pancreatic enzyme flows back into the bile, leading to bile duct carcinogenesis. Although the biliary tract resection and reconstruction is well documented to decrease the rate of malignancy, cancer occurrence has been reported in the residual intrahepatic or intrapancreatic bile duct, even after resection. We report a case of multiple biliary tract cancers in the liver complicated by congenital biliary dilatation, whose tumor lesions were resected en bloc without disconnecting the biliary tract by simultaneous pancreatoduodenectomy and living donor liver transplantation.

**Case presentation:**

A 27-year-old woman presented with epigastric discomfort. Examination indicated multiple biliary tract cancers complicated by congenital biliary dilatation. Computed tomography scan revealed three papillary tumors in the right hepatic duct with increased ^18^F-FDG accumulation on positron emission tomography. Contrast-enhanced ultrasound revealed another lesion in the left hepatic duct. Adenocarcinoma cells were detected using bile and choledochal brush cytology. Tumors resection by right lobectomy or trisegmentectomy of the liver and extrahepatic bile duct resection indicated a high risk of postoperative liver failure; the residual liver volumes were calculated only 277 ml or 176 ml, respectively. In addition, tumor recurrence owing to bile leakage during the surgery and carcinogenesis from the remaining bile duct were concerned. Pancreatoduodenectomy was performed without disconnecting the biliary tract, and the tumors were resected en bloc with the whole liver. The left lobe liver graft from the husband was then transplanted. After 5 years of adjuvant treatment with tegafur/gimeracil/oteracil potassium, she remained in remission eight and half years after the surgery.

**Conclusions:**

Given the mechanism and development of cancer in the congenital biliary dilatation, simultaneous pancreatoduodenectomy and liver transplantation may be considered, especially in the case of young patients.

## Background

In patients with pancreaticobiliary maljunction complicated by congenital biliary dilatation (CBD), the main pancreatic duct merges with the common bile duct on the liver side of the papilla of Vater [1], which allows pancreatic juice to reflux into the biliary tract [2]. The pancreatic enzyme that flows back into the bile duct is activated by enterokinase in the bile, causing genetic abnormalities by repeated damage and regeneration of the biliary epithelium [3]. This can result in hyperplastic changes with increased cell proliferation activity and, consequently, oncogene and/or tumor suppressor gene mutations in the epithelia, leading to bile duct carcinogenesis [2]. The prevalence of biliary tract cancers in adult pancreaticobiliary maljunction patients with biliary dilatation was reported to be 21.6% [4]. The rate of cholangiocarcinoma increases with age, and biliary tract cancers develop 15–20 years earlier than those without pancreaticobiliary maljunction [2, 5]. Patients discovered in their 20 s have a 2.3% risk of concomitant malignancy, which increases up to 75% in their 80 s [5].

The standard treatment for patients with CBD is biliary tract resection and reconstruction [6]. This treatment is well documented to decrease the rate of malignancy [7]. As the benefit of reducing the risk of cholangiocarcinoma outweighs the risk of surgery before the development of symptoms, surgical resection is recommended when diagnosed [6, 8]. However, cancer occurrence has been reported in the residual intrahepatic or intrapancreatic bile duct, even after resection [9]. The treatment concept must be shifted from prophylactic to curative surgery for patients who have already developed cholangiocarcinoma when diagnosed with CBD. Despite aggressive treatment with sufficient surgical margins, including lymphadenectomy, intrahepatic cholangiocarcinoma shows poor outcomes with a 5‐year survival rate of approximately 24% [10].

Here, we report a case of multiple biliary tract cancers in the liver complicated by CBD, whose tumor lesions were resected en bloc without disconnecting the biliary tract by simultaneous pancreatoduodenectomy and living donor liver transplantation (LTx).

## Case presentation

**Fig. 1:**
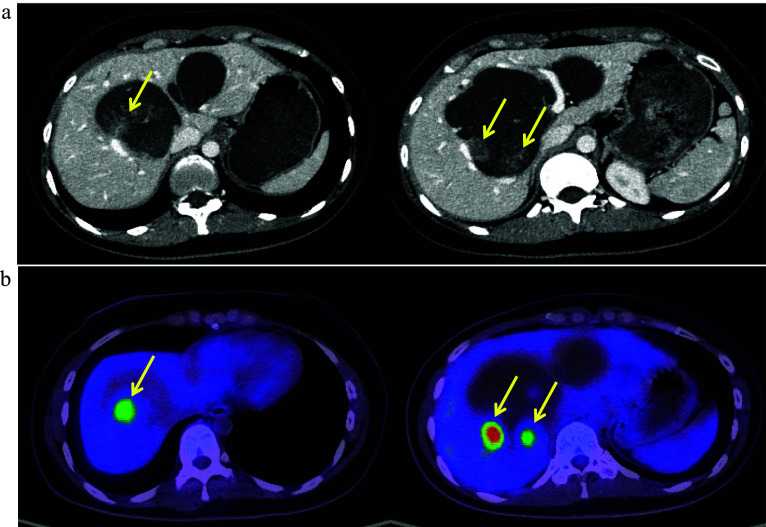
CT scan and FDG-PET. CT scan revealed three papillary tumors in the right hepatic duct (17, 21, and 11 mm from the left), and these lesions showed increased ^18^F-FDG accumulation (SUV_max_ = 14.1) on positron emission tomography (arrows). Neither lymphadenopathy nor distant metastasis was detected (**a** CT scan, **b** FDG-PET)

**Fig. 2:**
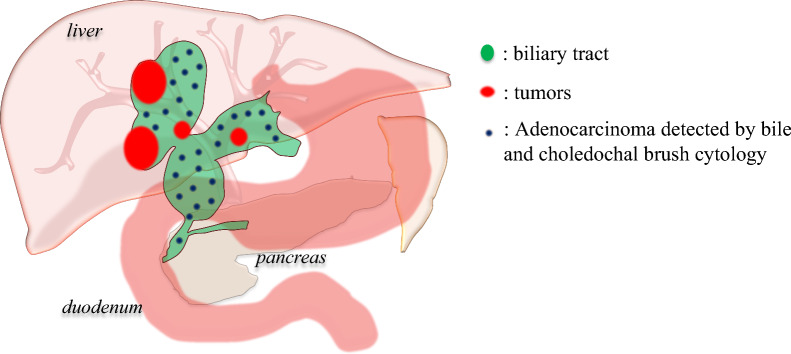
Overview of the biliary tract and tumors location. Three papillary tumors were located in the right hepatic duct and another lesion was also detected in the left hepatic duct. Adenocarcinoma cells were detected using bile and choledochal brush cytology on the common bile duct

A 27-year-old woman was referred to our clinic because of epigastric discomfort. Close examination indicated multiple biliary tract cancers complicated by CBD. Laboratory data showed slightly elevated serum gamma-glutamyl transpeptidase (55 U/L) and transaminase levels (aspartate transaminase: 43 U/L and alanine transaminase: 90 U/L); however, no tumor markers were elevated (alpha-fetoprotein: 1.8 ng/ml, carbohydrate antigen 19–9: 35.8 U/ml, and carcinoembryonic antigen: 2.7 ng/ml). The Child–Pugh and MELD scores were 5 and 7, respectively. Magnetic resonance cholangiopancreatography revealed that the main pancreatic duct merged with the bile duct at a high level. The cystic dilated bile duct was recognized continuously from the common bile duct to the hepatic ducts (Todani classification IV-A [11]). CT scan indicated the presence of three papillary tumors in the right dilated hepatic duct, which showed increased 18^F^-FDG accumulation on positron emission tomography (PET). Contrast-enhanced ultrasound (US) revealed another lesion in the left hepatic duct. Neither lymphadenopathy nor distant metastasis was detected (Fig. 1). Adenocarcinoma was confirmed using both bile and choledochal brush cytology from the intrapancreatic bile duct (Fig. 2). Tumor resection by hepatectomy and extrahepatic bile duct resection could not exclude the possibility of tumor recurrence owing to residual tumors and/or bile leakage during the surgery, and carcinogenesis from the remaining biliary tract was a major concern. Furthermore, right lobectomy and trisegmentectomy of the liver indicated a high risk of postoperative liver failure based on the liver volumetric evaluation; the residual liver volumes were calculated only 277 ml or 176 ml, respectively. Eventually, simultaneous pancreatoduodenectomy together with the whole hepatectomy and living donor LTx was selected to achieve complete removal of lesions without leaking bile, hoping for long-term survival and without tumor recurrence. Regarding neoadjuvant chemotherapy, any evidence-based preoperative chemotherapy for biliary cancer (complicated with CBD) was not established. Further, neither lymphadenopathy nor distant metastasis was detected at the time of diagnosis. Considering these clinical backgrounds, neoadjuvant chemotherapy which might have resulted in missing the opportunity for curative surgery, was not selected.

**Fig. 3:**
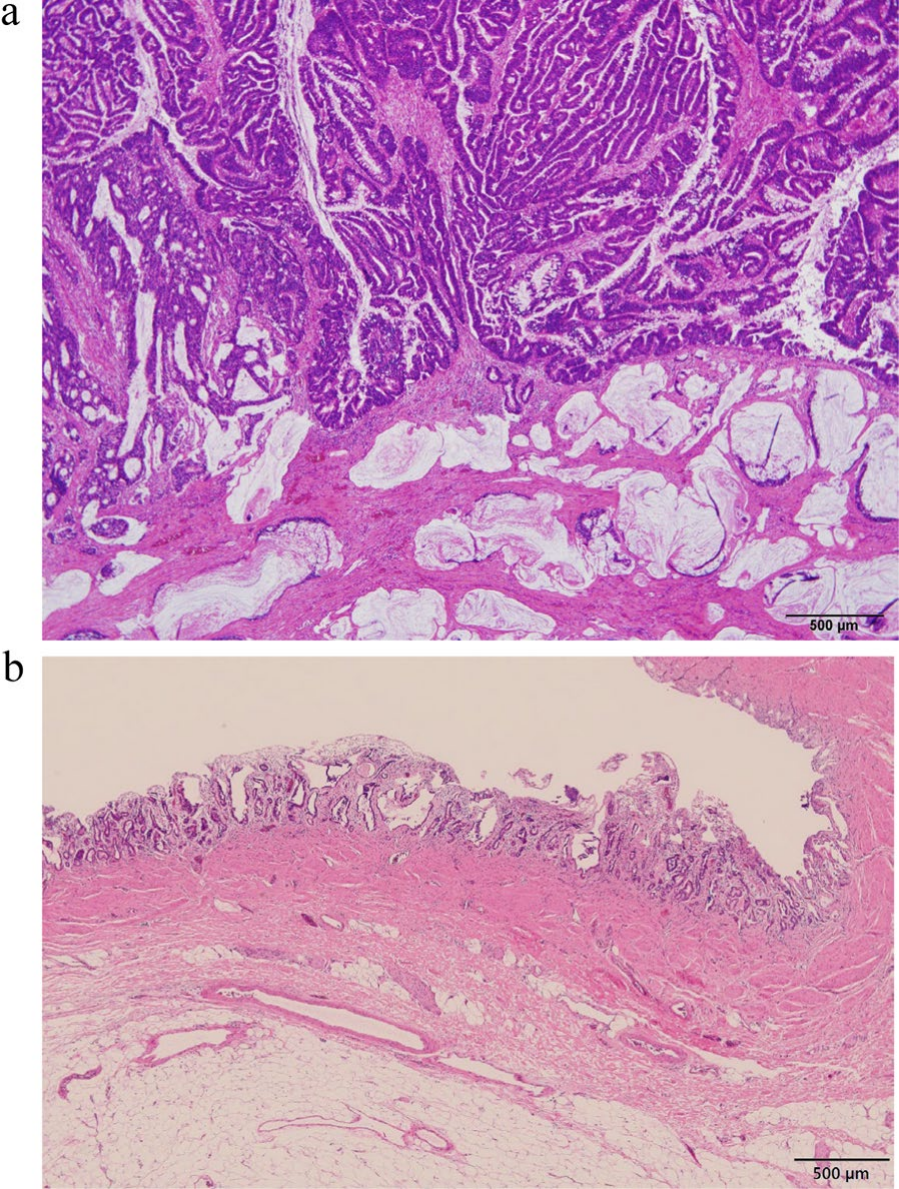
Pathological findings. Histopathologically, adenocarcinoma was located mainly in the right hepatic and common bile ducts (innumerable tumors, maximum diameter: 5.6 cm, papillary-infiltrating type, papillary adenocarcinoma > moderately differentiated tubular adenocarcinoma > mucinous adenocarcinoma). No vascular invasion was observed. Lymphatic metastasis was observed in a lymph node on the posterior surface of the pancreatic head (pathologically minimal lymphatic invasion). **a** The tumor invaded the bile duct wall and the liver parenchyma in some areas. **b** Biliary intraepithelial neoplasia was also observed in the left hepatic duct. Pathological diagnosis: pT2b, pN1, M0, Stage IIIc (UICC, 8th edition)

After confirming negative peritoneal lavage cytology, pancreatoduodenectomy and total hepatectomy with lymph node dissection (posterior of the head of the pancreas and hepatoduodenal ligament area) were performed without disconnecting the biliary tract. Then, living donor LTx was performed using the left liver graft donated by her husband. After the liver graft implantation (the left portal vein of the liver graft was anastomosed to the recipient main portal trunk, and the left hepatic artery of the liver graft was anastomosed to the recipient left hepatic artery), reconstruction was performed using the Whipple procedure. Pathologically, the main pancreatic duct merged with the common bile duct outside the duodenal wall, diagnosed as a pancreaticobiliary maljunction complicated by CBD. The adenocarcinoma lesions were located mainly in the right hepatic duct and expanded to the common bile duct, which partially invaded the liver parenchyma. Biliary intraepithelial neoplasia was also observed in the left hepatic duct (Fig. 3).

The postoperative course was uneventful. Methylprednisolone and basiliximab were given as an induction immunosuppressive therapy, and tacrolimus, mycophenolate mofetil, and low-dose methylprednisolone were used thereafter. The patient was discharged 3 weeks after the surgery. She received adjuvant chemotherapy with tegafur/gimeracil/oteracil potassium (TS-1) for 5 years to reduce the risk of recurrence as much as possible, considering the risk of carcinogenesis in immunosuppressed state. She remained in remission without tumor recurrence for eight and half years after the surgery.

## Discussion

LTx has been a treatment option for patients with hepatocellular carcinoma (HCC) who meet the indication criteria, addressing the widest surgical margin as well as recurrence risk factors by removing the damaged liver [12, 13]. By contrast, intrahepatic cholangiocarcinoma has been generally considered a contraindication for LTx due to a high tumor recurrence rate and poor outcomes. Meanwhile, several groups have reported acceptable outcomes after LTx in carefully selected intrahepatic cholangiocarcinoma patients [14]. Rea et al. reported that LTx combined with neoadjuvant chemoradiation achieved an outcome with an 82% 5-year survival rate in patients characterized with localized lesions without lymph node metastasis [15]. Sapisochin et al. also reported that patients with early intrahepatic cholangiocarcinoma had outcomes similar to those with HCC within the Milan criteria, defined as a single lesion ≤ 2 cm found on explant pathology [16]. However, these reports were conducted in a population without pancreaticobiliary maljunction. Cholangiocarcinoma in the CBD should be considered differently because carcinogenesis of the biliary tract accompanying pancreaticobiliary maljunction is associated with the hyperplasia–dysplasia–carcinoma sequence induced by chronic inflammation in the biliary duct, which differs from the adenoma–carcinoma sequence or the de novo carcinogenesis associated with biliary tract cancers in the population without pancreaticobiliary maljunction [17].

Therefore, in the present case, tumor extension and disease characteristics prompted us to consider whole hepatectomy with pancreatoduodenectomy followed by LTx as the best treatment option to achieve long-term survival. Although several cases have been reported in which long-term survival was achieved by simultaneous liver transplantation and pancreaticoduodenectomy for cholangiocarcinoma [18–23], to our knowledge, this is the first report of simultaneous pancreatoduodenectomy and living donor LTx for biliary cancer complicated by CBD. However, life-long immunosuppressive therapy after LTx accelerates the recurrence of circulating cancer cells and/or micrometastases. As well as adjuvant treatment with TS-1, minimal immunosuppressive therapy and switching from CNIs to mTORi which has recently become available, would hopefully contribute to minimizing the risk of tumor recurrence. Although the patient remained in remission eight and half years after the surgery, comprehensive follow-up is still required.

## Conclusions

From the transplant oncology point of view, simultaneous pancreatoduodenectomy and LTx may be considered especially in young patients, given the mechanism and development of cancer in the CBD.

## Data Availability

Data will be made available on reasonable request.

## Abbreviations

CBD: Congenital biliary dilatation
CT: Computed tomography
HCC: Hepatocellular carcinoma
LTx: Liver transplantation
PET: Positron emission tomography
TS-1: Tegafur/gimeracil/oteracil potassium
US: Ultrasound
